# Multidimensional Recovery Trajectories Throughout Breast Cancer Treatment and Survivorship: A Prospective Longitudinal Study

**DOI:** 10.3390/curroncol33090567

**Published:** 2026-09-19

**Authors:** Laura Lorenzo-Gallego, Nicola S. Diciolla, Fernando Ramos-Gómez, Virginia Prieto-Gómez, Aldina Couso-González, María Torres-Lacomba

**Affiliations:** 1 Physiotherapy Department, Physiotherapy in Women’s Health Research Group, Faculty of Physiotherapy, University of Alcalá, 28805 Alcalá de Henares, Madrid, Spain; laura.lorenzo@uah.es (L.L.-G.); v.prieto@uah.es (V.P.-G.); aldina.couso@uah.es (A.C.-G.); maria.torres@uah.es (M.T.-L.); 2 Ramón y Cajal Institute of Health Research—IRYCIS, University Hospital of Ramón y Cajal, 28034 Madrid, Spain; 3 Respiratory Research and Rehabilitation Laboratory—Lab3R, School of Health Sciences—ESSUA, University of Aveiro, 3810-193 Aveiro, Portugal; 4 Institute of Biomedicine–IBiMED, University of Aveiro, Campus Universitário de Santiago, Agras Do Crasto, 3810-193 Aveiro, Portugal; 5 Department of Physiotherapy, Medicine and Biomedical Sciences, Faculty of Physiotherapy, University of A Coruña, 15006 A Coruña, Spain; fernando.ramos@udc.es; 6 Department of Obstetrics and Gynecology, Príncipe de Asturias University Hospital, 28805 Alcalá de Henares, Madrid, Spain

**Keywords:** breast cancer, cancer survivorship, rehabilitation, quality of life, quantitative sensory testing, patient-reported outcomes, supportive care

## Abstract

**Background**: Breast cancer survivors frequently experience persistent sensory, psychosocial, and functional sequelae that may not be captured by pain measures alone. Understanding recovery trajectories throughout treatment and survivorship may help identify women at risk of long-term recovery-related difficulties. **Objective**: To investigate sensory, psychosocial, functional, and quality-of-life trajectories throughout breast cancer treatment. **Methods**: This prospective longitudinal study included women undergoing unilateral breast cancer surgery assessed at six time points before and after surgery, including before and after adjuvant treatments when applicable. Outcomes included sensory function, pain, upper-limb function, psychosocial variables, and quality of life. **Results**: Seventy-two women (57 ± 11 years) were included. Despite stable pain intensity and self-reported sensitization symptoms, fear of movement increased (TSK-11: MD [95%CI] = +9[0;22]), quality of life deteriorated (FACT-B: 3–12 month MD [95%CI] = −10[−19;−1]), and upper-limb function initially worsened before partially recovering. Objective sensory alterations suggested dynamic changes in somatosensory processing but were not consistently accompanied by greater pain intensity or self-reported sensitization symptoms. **Conclusions**: Recovery after breast cancer treatment is multidimensional and cannot be adequately reflected by pain intensity alone. Multidimensional assessment may help identify women at risk of persistent recovery-related difficulties and support personalized survivorship care.

## Simple Summary

Many women continue to experience physical, sensory, and emotional problems after breast cancer treatment, even when pain levels appear to improve. Understanding how recovery evolves over time is essential to provide appropriate follow-up and support during survivorship. In this prospective longitudinal study, women were assessed before surgery and throughout treatment using clinical, sensory, functional, and quality-of-life measures. Recovery was not uniform across these domains: pain remained relatively stable, whereas fear of movement increased, quality of life worsened, and objective sensory changes persisted over time. These findings show that pain intensity alone does not adequately reflect recovery after breast cancer treatment. A multidimensional assessment may help clinicians identify women at risk of persistent recovery-related difficulties and support more personalized survivorship care.

## 1. Introduction

Breast cancer is the most frequently diagnosed cancer among women worldwide, accounting for 11.6% of new cancer cases and 6.9% of cancer-related deaths [1]. Advances in early detection and treatment have substantially improved survival [2,3], resulting in a growing population of breast cancer survivors. However, many women continue to experience persistent treatment-related sequelae that may affect physical function, participation, psychosocial well-being and health-related quality of life (HRQoL) long after completion of treatment [4,5]. Persistent pain affects up to 60% of breast cancer survivors and remains one of the most common long-term sequelae of cancer treatment [4].

Recovery after breast cancer treatment is complex and multidimensional. Oncological treatments may contribute to persistent symptoms through multiple biological, physical and psychosocial mechanisms [6,7,8,9]. Surgical treatment may contribute to musculoskeletal pain, joint dysfunction and persistent postsurgical pain, which affects approximately 50% of women and is moderate-to-severe in up to 25% [8,10]. Radiotherapy and chemotherapy may further contribute to sensory disturbances, neuropathic symptoms and altered pain modulation through inflammatory and neural mechanisms [8,11,12]. Hormonal therapies such as aromatase inhibitors may also contribute to musculoskeletal pain and altered pain perception [13,14]. Additional factors including obesity, low educational level and lymphedema may further influence symptom persistence and recovery [6]. Up to 30% of survivors report pain a decade after treatment [6], with substantial consequences for function, psychosocial well-being and HRQoL. The latter is particularly relevant as a multidimensional construct encompassing physical, psychological and social aspects of health and functioning [15]. The broader importance of quality-of-life assessment is reflected in multidimensional instruments developed by the World Health Organization, such as the WHOQOL-BREF, which assesses physical, psychological, social and environmental domains [16]. Psychosocial factors such as anxiety, distress, depression and catastrophizing may further influence symptom persistence and recovery trajectories [17,18]. Collectively, these treatment-related sequelae may contribute to heterogeneous survivorship trajectories and ongoing supportive care needs.

Pain-related symptoms after breast cancer treatment are multidimensional and may involve nociceptive, neuropathic and nociplastic mechanisms [19,20]. Neuropathic and nociplastic pain have been associated with poorer HRQoL and psychosocial functioning [21]. Central and peripheral sensitization may contribute to altered pain processing and pain chronification [22,23,24,25]. Nevertheless, pain intensity alone may not adequately capture the multidimensional nature of recovery after breast cancer treatment. A more comprehensive understanding of sensory, psychosocial and functional recovery patterns may help identify women at risk of persistent difficulties and support more personalized survivorship care.

Quantitative sensory testing (QST) provides an objective approach to characterizing sensory alterations that may accompany breast cancer treatment and survivorship [26,27,28]. Previous studies have identified sensory abnormalities at different stages of treatment and recovery [29,30,31,32,33,34,35]. However, most available evidence is cross-sectional, focused on isolated treatment stages, or based on partial sensory assessment protocols, limiting understanding of how sensory, psychosocial, functional and quality-of-life outcomes evolve longitudinally throughout the breast cancer continuum. Thus, longitudinal studies integrating these different dimensions across the treatment continuum remain limited. Understanding these trajectories may facilitate identification of women at risk of persistent recovery-related difficulties and unmet survivorship needs.

Therefore, this study aimed to investigate longitudinal sensory, psychosocial, functional and health-related quality-of-life trajectories during and after breast cancer treatment using quantitative sensory testing alongside patient-reported outcomes related to pain, upper-limb function, psychosocial factors and HRQoL.

We hypothesized that women undergoing breast cancer treatment would exhibit time-dependent sensory and psychosocial changes across the recovery trajectory and that objective changes in sensory processing would not necessarily parallel pain intensity, self-reported sensitization symptoms or other patient-reported outcomes.

## 2. Materials and Methods

### 2.1. Design

A prospective observational study was conducted at the Physiotherapy “BLINDED” Research Unit “BLINDED” to investigate longitudinal sensory, psychosocial, functional and HRQoL trajectories across breast cancer treatment and survivorship. All participants provided written informed consent. Study procedures complied with the ethical standards of the institutional and national research committees and with the Declaration of Helsinki. Data were pseudonymized according to European data protection regulations. The study was approved by the Research Ethics Committee of Hospital Universitario Príncipe de Asturias (protocol code OE 16/2019; approved 4 November 2019). The study was reported according to the Strengthening Reporting of Observational Studies in Epidemiology (STROBE) guidelines [36]. As this was an observational cohort study, clinical trial registration was not applicable.

### 2.2. Participants

Women with unilateral breast cancer scheduled for surgery with axillary lymph node dissection or sentinel lymph node biopsy were consecutively recruited by a physician from the Gynaecology and Obstetrics Department at “BLINDED”. Exclusion criteria included neurological disorders, previous shoulder surgery or severe upper-limb conditions, cognitive impairment, and receipt of oncological treatment within the previous year. All participants provided written informed consent before enrolment.

All participants underwent breast cancer surgery. Additionally, 15 participants received chemotherapy and 34 participants received radiotherapy, with partial overlap between treatment modalities. Chemotherapy and radiotherapy were administered according to standard clinical protocols and initiated after surgery. The timing and duration of adjuvant treatments varied across individuals, reflecting routine clinical practice.

### 2.3. Assessment

**Figure 1:**
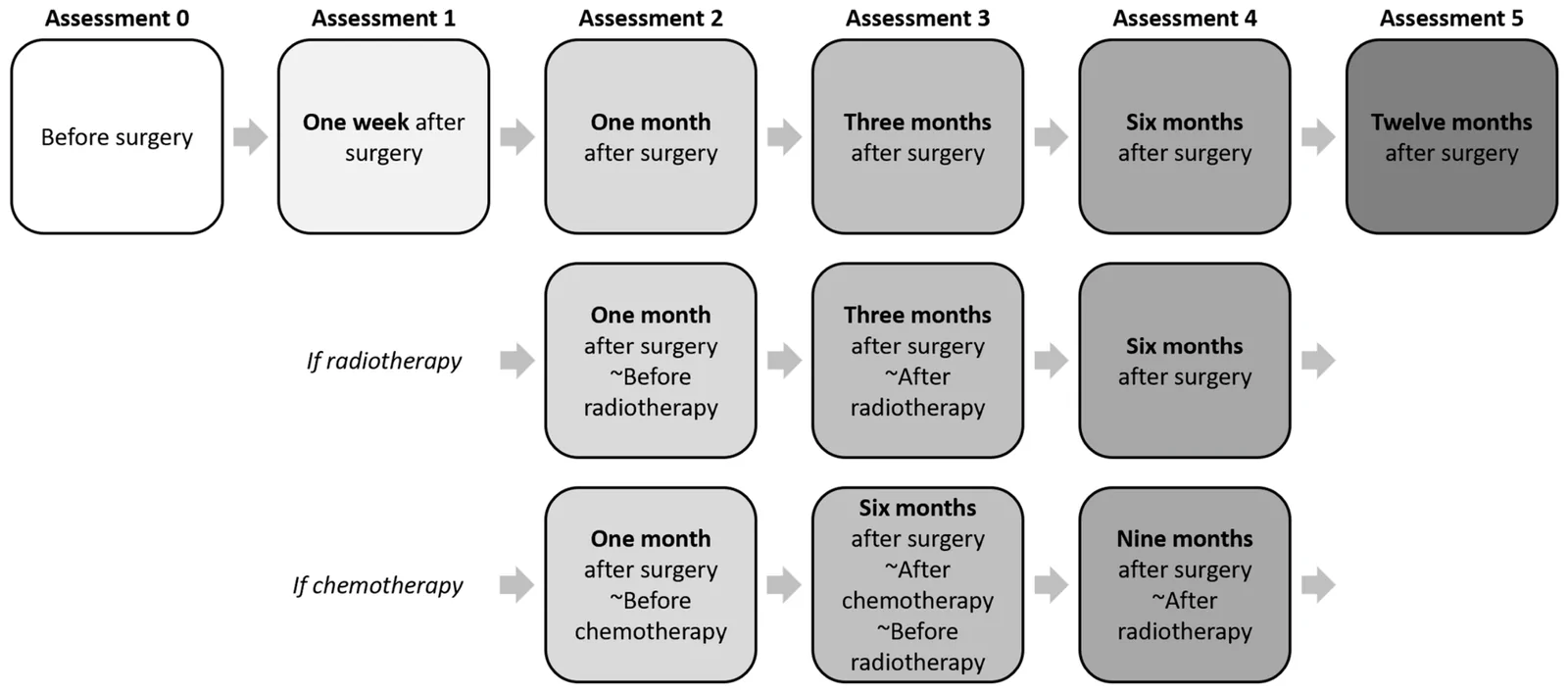
Assessment schedule across breast cancer treatment and follow-up. Seventy-two participants were enrolled at baseline before surgery. Assessments were conducted at six predefined time points aligned with key clinical milestones: before surgery (Assessment 0), one week after surgery (Assessment 1), and follow-up assessments approximately one (Assessment 2), three (Assessment 3), six (Assessment 4), and twelve months (Assessment 5) after surgery. These assessment time points represent predefined clinical assessment phases and were modelled as such in the statistical analyses, rather than as fixed calendar intervals. For participants receiving adjuvant treatments, Assessments 2–4 coincided with periods before and after chemotherapy and/or radiotherapy, when applicable, depending on individual clinical pathways. Not all assessment points applied to all participants. Note: ~ indicates approximate timing in relation to oncological treatment.

Assessments were conducted at six predefined time points corresponding to key milestones across the breast cancer treatment trajectory (Figure 1), selected to capture the preoperative period, acute and early postoperative recovery, adjuvant-treatment phases, and longer-term survivorship. This assessment schedule is consistent with the expected sequencing of oncological treatments [2,37], established longitudinal outcome assessment frameworks in breast cancer [38], and previous prospective evidence on postsurgical pain trajectories [39]. Assessments were scheduled according to predefined clinical phases (i.e., 1 week before surgery, 1 week after surgery, and approximately 1, 3, 6, and 12 months after surgery); however, variability in actual calendar timing occurred, particularly during the adjuvant-treatment phases (A3–A5), depending on individual treatment pathways. Accordingly, not all assessment points had the same clinical meaning across participants, and intervals between assessments differed across individuals. The actual timing of assessments relative to surgery is reported in Supplementary Table S1. Therefore, assessment points should be interpreted as nominal clinical follow-up phases rather than identical calendar-time points across participants.

At baseline, sociodemographic characteristics were collected. During follow-up, anthropometric variables (weight, height, body mass index [BMI], menopausal status) and clinical variables (upper-limb history, affected side, surgery type, complications such as seroma, superficial lymphatic thrombosis and acute pain, adjuvant treatments, and breast reconstruction) were recorded.

Information regarding chemotherapy (e.g., anthracyclines, taxanes), hormone therapy (e.g., tamoxifen, aromatase inhibitors) and biological therapy (e.g., trastuzumab, CDK4/6 inhibitors) was collected throughout follow-up. Analgesic or pain medication use was not systematically recorded because few participants reported clinically relevant pain at baseline and medication use was not routinely documented during follow-up.

Pain onset, location, and intensity, functional impact, sensory descriptors, psychosocial variables (pain catastrophizing, kinesiophobia and pain acceptance), HRQoL and pain sensitization were assessed at each time point. Pain sensitization was evaluated using complementary objective and self-report measures as part of a multidimensional assessment of recovery.

### 2.4. Outcome Measures

#### 2.4.1. Objective Sensory Assessment

QST followed the standardized protocol proposed by Rolke et al. [27] and adapted for breast cancer populations [40]. The protocol included mechanical detection threshold (MDT), mechanical allodynia, vibration detection threshold (VDT), thermal perception and pain thresholds, temporal summation (TS), pressure pain threshold (PPT), supra-threshold pressure stimulation, and conditioned pain modulation (CPM).

#### 2.4.2. Mechanical Detection Threshold

Von Frey filaments (Aesthesiometer, Stoelting Co., Wood Dale, IL, USA) ranging from 0.23 to 512 mN were applied in ascending and descending order to the medial third of the humerus bilaterally. Participants indicated the presence or absence of tactile sensation. Painful responses were classified as allodynia.

#### 2.4.3. Vibration Detection Threshold

VDT was assessed using a Rydel–Seiffer 64 Hz tuning fork (Valuemed^®^, Edmonton, AB, Canada) applied bilaterally to the epicondyle, radial styloid and acromion. VDT was operationalized and recorded as vibration decay time (seconds), consistent with the device output and previous studies.

#### 2.4.4. Thermal Detection and Pain Thresholds

Cold (25 °C) and heat (40 °C) stimuli (Rolltemp II, Somedic SenseLab AB, Sösdala, Sweden) were applied bilaterally to the posterior deltoid, serratus anterior and rectus abdominis muscles. Presence or absence of thermal sensation and pain intensity were recorded.

#### 2.4.5. Temporal Summation

Punctate stimuli (256 mN) were applied bilaterally to the mid-deltoid. Pain ratings were recorded following a single stimulus and after 10 consecutive stimuli delivered at one stimulus per second, repeated five times. The wind-up ratio (WUR) was calculated by dividing the mean pain rating following repeated stimuli by the mean pain rating following single stimuli. WUR values > 1 indicated facilitation, values = 1 no change, and values < 1 reduced summation [41]. WUR values > 1 were not considered indicative of a clinically abnormal response, as no universal clinical cut-off has been established and interpretation of abnormality requires consideration of appropriate normative reference data and the assessment methodology [27,41,42].

#### 2.4.6. Pressure Pain Threshold

PPT was assessed on the non-affected side using a handheld algometer (Wagner Instruments, Greenwich, CT, USA) at the serratus anterior, middle scalene, lateral epicondyle tendon and vastus lateralis. Three measurements were obtained at each site and averaged, with 30 s rest periods between assessments.

##### Supra-Threshold Pressure Stimulation

A stimulus corresponding to 120% of the PPT was applied using the algometer to the infraspinatus on the non-affected side. Pain location and associated sensations were recorded using a body map [43].

#### 2.4.7. Conditioned Pain Modulation

CPM was evaluated using a submaximal effort tourniquet test performed on the contralateral arm [22,44]. PPTs were first assessed at the infraspinatus (bilaterally) and contralateral quadriceps. Following one minute of arm elevation, a cuff was inflated to 270 mmHg and participants performed wrist extensions using a 1 kg weight. Pain intensity was assessed using an 11-point numerical rating scale (NRS, 0–10). After 20 repetitions, participants reported pain intensity; if pain remained below 3 points, the task continued up to a maximum of 45 repetitions or until pain intensity reached ≥3. The final NRS value achieved during the conditioning task was recorded. Immediately after the conditioning stimulus, PPTs were reassessed at the same sites. The CPM effect was calculated as the difference between post-conditioning and pre-conditioning PPT values (ΔPPT = post-PPT minus pre-PPT), with lower ΔPPT values indicating reduced endogenous pain inhibition.

#### 2.4.8. Patient-Reported Outcomes

Participants completed a battery of validated self-report instruments.

Central sensitization-related symptoms were assessed using the Spanish version of the Central Sensitization Inventory (CSI) [45].

Psychological variables relevant to pain sensitization and survivorship outcomes included pain catastrophizing, measured using the Pain Catastrophizing Scale (PCS) [46,47], fear of movement, evaluated using the Tampa Scale of Kinesiophobia (TSK-11) [48], and pain acceptance, assessed using the Chronic Pain Acceptance Questionnaire (CPAQ) [49], selected based on their relevance to pain-related psychological responses and their previous use in cancer populations [50,51,52].

HRQoL was assessed using the Functional Assessment of Cancer Therapy-Breast (FACT-B, version 4), a breast cancer-specific instrument validated in Spanish women, which evaluates physical, emotional, social and functional well-being, together with breast cancer-specific and arm-related symptoms [53].

##### Pain and Upper-Limb Function

When present, pain location, intensity and impact were assessed using the Brief Pain Inventory (BPI) [54,55], the Shoulder Pain and Disability Index (SPADI) [56], and the Self-report Leeds Assessment of Neuropathic Symptoms and Signs (S-LANSS) [57]. For longitudinal analyses, the BPI pain severity score (mean of worst, least, average and current pain; range 0–10) was used.

#### 2.4.9. Statistical Analysis

Sample-size estimation was based on expected within-subject longitudinal changes in pain sensitivity measures derived from CPM. Based on previously reported mean differences in pain intensity (2.3 ± 3.6 points on the numerical rating scale in the infraspinatus muscle) and PPT (−13.33 ± 19.12 N in the infraspinatus muscle), effect sizes ranging from 0.68 to 0.74 were estimated [58]. Assuming α = 0.05 and 95% statistical power, a minimum sample size of 38–44 participants was required. Anticipating a 40% dropout rate, a target sample size of 53–62 participants was established. Seventy-two participants were eventually enrolled; this sample size was computed specifically for CPM/PPT-related outcomes and therefore was not intended to provide outcome-specific power for other exploratory QST and patient-reported outcomes.

Descriptive statistics were calculated for all variables. Continuous variables are presented as mean ± standard error or median (Q1;Q3), depending on distributional assumptions assessed using the Shapiro–Wilk test, visual inspection of histograms and Q-Q plots, and skewness and kurtosis indices. Categorical variables are presented as absolute and relative frequencies.

Longitudinal changes in continuous outcomes across the six assessment points were analysed using linear mixed-effects models with restricted maximum likelihood estimation. Time was modelled as a fixed within-subject effect and participant as a random intercept. Assessment was modelled as a categorical within-participant factor representing predefined clinical follow-up phases rather than exact calendar time. Therefore, the model estimates average changes across these nominal assessment phases and should not be interpreted as representing homogeneous treatment-specific trajectories. Models were prespecified to adjust for age, baseline BMI, number of chemotherapy cycles and number of radiotherapy sessions. Mixed-effects models allowed inclusion of all available observations under the missing-at-random assumption, without restricting analyses to participants with complete follow-up data [59].

To explore potential attrition-related differences, baseline characteristics and outcome measures were compared descriptively between participants who completed the 12-month assessment and those who did not (i.e., completers and non-completers). An estimation-based approach was used. For approximately normally distributed continuous variables, unadjusted mean differences (non-completers minus completers) and 95% bias-corrected and accelerated bootstrap confidence intervals were computed using 5000 bootstrap resamples. For non-normally distributed continuous variables, Hodges–Lehmann estimates of the between-group location shift and 95% confidence intervals were calculated.

Results are presented as estimated marginal means ± standard errors, estimated mean differences and adjusted 95% confidence intervals.

For outcomes that did not satisfy model assumptions despite transformation attempts, complementary Friedman tests were performed. Repeated categorical outcomes were analysed using Cochran’s Q test.

As there were several multidimensional outcome measures, analyses were classified as primary/confirmatory or exploratory. Primary QST measures, i.e., PPT- and CPM-related measures underpinning the a priori sample-size computation, constituted the principal outcome family. For this principal outcome family, family-wise Type I error across omnibus longitudinal tests was controlled using the Holm procedure, with Holm-adjusted *p* < 0.05 considered statistically significant. The remaining QST and patient-reported outcome measures were considered exploratory; their omnibus *p*-values are reported unadjusted and interpreted as hypothesis-generating rather than according to a formal significance threshold. Where post hoc pairwise comparisons were performed, *p*-values were adjusted using the Bonferroni procedure, with Bonferroni-adjusted *p* < 0.05 considered statistically significant.

Subgroup analyses according to treatment modality were not performed because the study was not powered to detect between-group differences. Treatment exposure was instead incorporated through covariate adjustment within the longitudinal models.

All analyses were performed using IBM SPSS Statistics version 29 (IBM Corp., Armonk, NY, USA).

## 3. Results

### 3.1. Participant Characteristics

**Figure 2:**
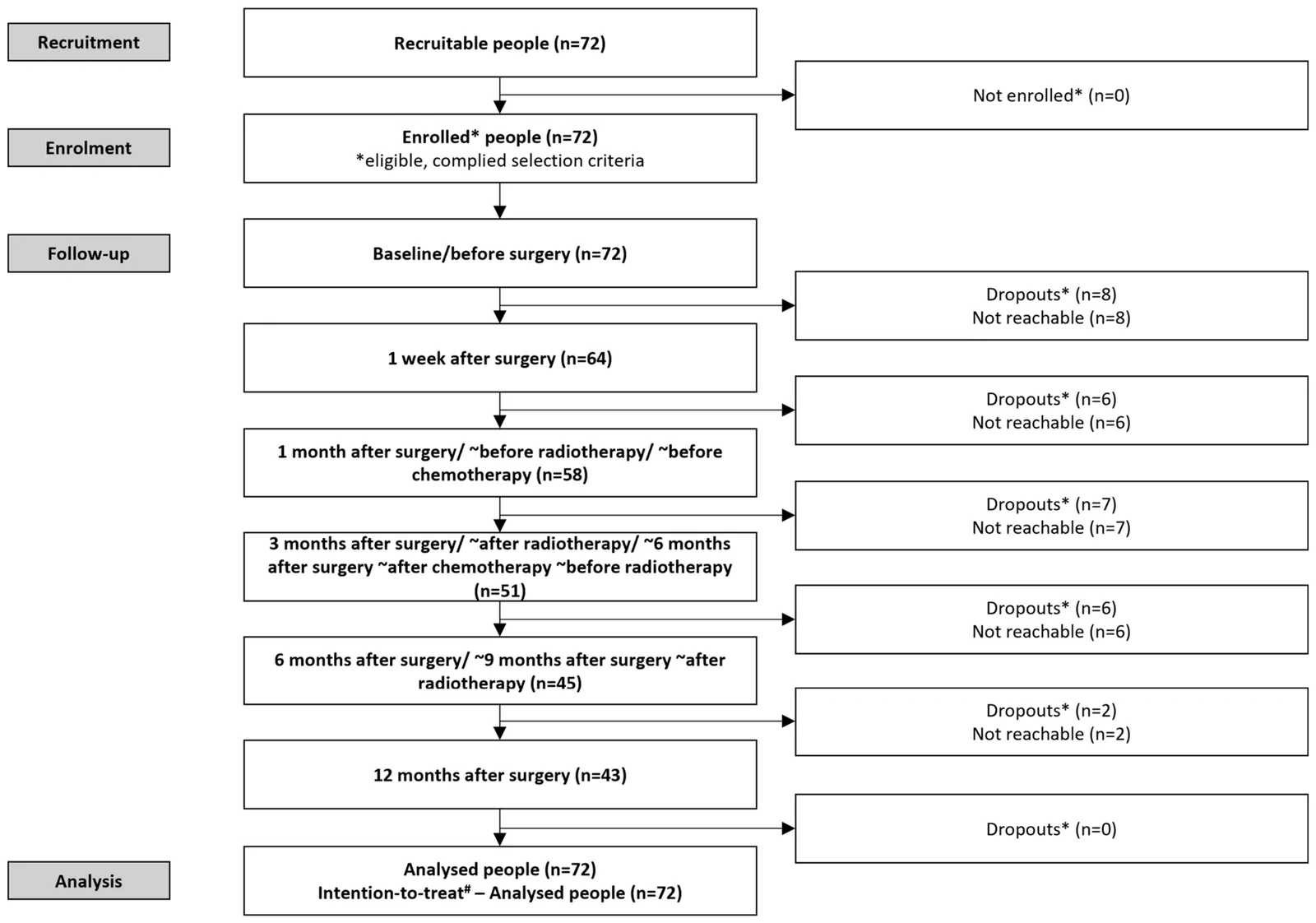
Flow diagram of participant recruitment, follow-up, and inclusion in longitudinal analyses. Abbreviation: n = number of participants; ~ indicates approximate assessment timing.

Seventy-two women (mean age 57 ± 11 years; BMI 27.5 ± 5.6 kg·m^−2^) were enrolled, and 43 completed the 12-month follow-up assessment (Figure 2).

Baseline sociodemographic and clinical characteristics are presented in Table 1. All available observations were included in the longitudinal analyses using linear mixed-effects models. The actual timing of assessments relative to surgery is reported in Supplementary Table S1. Although assessments followed the predefined clinical schedule, variability in calendar timing was observed, particularly during the intermediate follow-up assessments, when participants followed different adjuvant-treatment pathways.

Forty-three participants completed the 12-month assessment and 29 did not. Baseline comparisons according to follow-up completion status are presented in Supplementary Table S2. Most between-group differences in quantitative sensory testing and psychosocial outcomes were small or imprecisely estimated. However, non-completers reported greater pain intensity during the conditioning stimulus than completers (NRS 0–10: MD[BCa95%CI] = +1.67[0.26;3.06]) and poorer baseline HRQoL (FACT-B total score: estimate [95%CI] = −13[−22;−4]; FACT-B TOI score: −7[−14;−2]; FACT-G total score: −13[−21;−4]).

### 3.2. Quantitative Sensory Testing and Sensory Processing

Quantitative sensory testing outcomes and longitudinal sensory trajectories are presented in Table 2A,B. Graphical representations of sensory trajectories are provided in the Supplementary Materials (Figures S1–S6).

Objective sensory changes were observed throughout follow-up. These changes were dynamic and heterogeneous and were not consistently reflected by pain severity or self-reported sensitization symptoms.

#### 3.2.1. Pressure Pain Sensitivity and Pain Modulation

PPTs exhibited muscle-specific and non-uniform changes over time (Table 2A). Significant overall time effects were observed in the scalene (A4-A2: MD [95%CI] = +0.53[0.03;1.03]; A4-A3: MD [95%CI] = +0.59[0.03;1.15]) and serratus anterior (A4-A1: MD [95%CI] = −0.94[−1.70;−0.17]; A4-A3: MD [95%CI] = −0.86[−1.67;−0.06]) muscles on the non-affected side, although post hoc analyses revealed heterogeneous patterns without a consistent directional trend across follow-up.

CPM responses changed progressively over time. Conditioning pain intensity increased significantly (A4-A0: MD [95%CI] = +4.12[0.24;8.01]; A5-A0: MD [95%CI] = +4.19[0.01;8.37]; main time effect, *p* = 0.031), while CPM responses at the rectus femoris progressively decreased, indicating reduced endogenous pain inhibitory capacity (Table 2A). Changes in CPM responses at the infraspinatus did not reach statistical significance.

For supra-threshold pressure stimulation, referred pain became less frequent over time (11% at baseline to 5% at 12 months), whereas pain intensity showed a non-significant decrease (Table 2A).

#### 3.2.2. Detection Thresholds

Mechanical detection thresholds, thermal detection thresholds, and thermal pain thresholds remained largely stable throughout follow-up (Table 2B). No significant longitudinal changes were observed for MDTs on either the affected or non-affected side, and no evidence of mechanical allodynia was identified at any assessment point.

In contrast, longitudinal changes were identified in selected quantitative sensory testing parameters (Table 2B), particularly vibration detection thresholds, temporal summation, conditioned pain modulation and selected pressure pain threshold measures.

VDT demonstrated site-specific changes over time. Overall time effects were observed at the acromion (A1-A0: MD [95%CI] = −3.35[−6.58;−0.13]), epicondyle (A4-A1: MD [95%CI] = +7.55[2.61;12.48]; A4-A2: MD [95%CI] = +6.58[3.29;9.88], A4-A3: MD [95%CI] = +4.26[0.08;8.44]) and radial styloid (A5-A2: +4.80[0.78;9.10]) on the affected side and at the epicondyle (A4-A1: MD [95%CI] = +8.33[1.71;14.97]; A4-A2: MD [95%CI] = +8.17[3.82;12.52]) and radial styloid (A3-A0: MD [95%CI] = +5.11[0.00;10.51]) on the non-affected side (all *p* < 0.01). On the affected side, VDT at the acromion showed a transient decline one week after surgery, whereas VDT values at the epicondyle and radial styloid increased during later follow-up assessments. Similar increases were observed at selected sites on the non-affected side, particularly during mid-to-late follow-up. These findings suggest evolving sensory changes throughout the recovery trajectory.

Temporal summation remained facilitated throughout follow-up, with WUR values consistently greater than 1 (Table 2B). An overall time effect was observed on the affected side (*p* < 0.01), characterized by an initial increase following surgery and a gradual decline thereafter. No clear longitudinal changes were identified on the non-affected side.

### 3.3. Recovery-Related Outcomes

#### 3.3.1. Pain, Psychosocial Variables and Health-Related Quality of Life

Pain, psychosocial variables, upper-limb function and HRQoL outcomes are summarized in Table 3.

Recovery trajectories differed across outcome domains. While pain intensity and self-reported sensitization symptoms remained relatively stable throughout follow-up, clinically relevant changes were observed in psychosocial outcomes, HRQoL and upper-limb function.

Pain intensity remained relatively stable across the study period, with no clear longitudinal changes in BPI pain severity scores (0[−1;1] points at 12 months after surgery from baseline; *p* = 0.255) (Table 3). Similarly, self-reported central sensitization symptoms, pain catastrophizing and pain acceptance did not demonstrate significant overall time effects (CSI: *p* = 0.86; PCS: *p* = 0.11; CPAQ: *p* = 0.34) (Table 3). The proportion of participants reporting severe pain remained low throughout follow-up (5%).

In contrast, kinesiophobia increased over time (main time effect, *p* < 0.002), rising +9[0;22] points at 12 months after surgery from baseline (Table 3). The largest increase was observed during the later stages of follow-up, suggesting persistent fear of movement despite the absence of worsening pain severity.

HRQoL also deteriorated throughout follow-up (FACT-B, main time effect, *p* < 0.001) (Table 3). The largest decline was observed between 3 and 12 months after surgery (MD [95%CI] = −10[−19;−1]), indicating a deterioration in perceived HRQoL during survivorship.

#### 3.3.2. Upper-Limb Function

Upper-limb function deteriorated transiently following surgery (Table 3). SPADI scores worsened during the early and mid-follow-up assessments before partially recovering at 12 months after surgery. Neuropathic pain-related symptoms (S-LANSS) increased modestly during mid-to-late follow-up without evidence of progressive worsening.

Overall, quantitative sensory testing revealed dynamic changes in somatosensory processing throughout the breast cancer continuum that were not consistently paralleled by pain severity or self-reported sensitization symptoms.

## 4. Discussion

This prospective observational study provides a multidimensional perspective on recovery after breast cancer treatment by integrating sensory, psychosocial, functional and HRQoL outcomes across the treatment continuum. Overall, the findings indicate that survivorship trajectories are heterogeneous and multidimensional, characterized by dynamic changes in sensory processing, fear of movement and quality of life that were not consistently reflected by pain severity alone. Notably, pain intensity remained relatively stable, whereas kinesiophobia increased and HRQoL deteriorated over time. These findings reinforce the importance of multidimensional survivorship assessment beyond pain intensity or isolated physical outcomes and support the integration of sensory, psychosocial and functional domains within breast cancer survivorship care.

Although upper-limb function declined initially after surgery, scores returned to near-baseline levels by the end of follow-up. In contrast, HRQoL decreased over time, suggesting that recovery after breast cancer treatment extends beyond restoration of physical function alone. The dissociation between relatively preserved upper-limb function and worsening HRQoL highlights the complexity of survivorship experiences and supports the use of multidimensional biopsychosocial assessment approaches. Psychological, social and emotional factors are likely to contribute to perceived well-being after treatment [60]. Furthermore, increased kinesiophobia has previously been associated with poorer HRQoL in breast cancer survivors [61], a pattern consistent with the trajectories observed in the present cohort. Deteriorated CPM responses may also be clinically relevant, as impaired endogenous pain inhibitory capacity has been associated with greater pain interference, poorer HRQoL and persistent survivorship-related difficulties in chronic pain populations [62]. Together, these findings suggest that survivorship challenges may persist despite partial physical recovery.

Psychosocial outcomes followed distinct trajectories throughout follow-up. While pain catastrophizing remained stable and pain acceptance showed little change, kinesiophobia increased significantly, particularly during the later stages of follow-up. From a survivorship and rehabilitation perspective, this finding may be especially relevant because fear of movement can contribute to activity restriction, reduced participation, avoidance behaviours and delayed recovery despite relatively stable pain intensity. Psychological distress of this kind has previously been associated with prolonged pain and poorer outcomes in breast cancer survivors [17,18,63]. Factors such as fatigue or lymphedema-related concerns have been associated with kinesiophobia in previous studies [64,65,66,67,68,69], but their contribution to the trajectory observed here cannot be established because they were not assessed in the present study. These findings reinforce the importance of incorporating psychosocial assessment and behavioural rehabilitation strategies within survivorship care.

Pain intensity remained moderate and relatively stable throughout follow-up, whereas neuropathic pain features fluctuated over time. Stable pain intensity should not necessarily be interpreted as the absence of ongoing recovery-related difficulties, as sensory alterations, psychosocial challenges and HRQoL impairments may persist despite relatively low symptom severity [30,70,71,72,73,74]. These findings suggest that reliance on pain intensity alone may underestimate the complexity of survivorship recovery.

Objective sensory changes were observed throughout follow-up despite relatively stable pain intensity and self-reported sensitization symptoms, suggesting that somatosensory recovery may evolve independently from subjective symptom severity in some breast cancer survivors. While mechanical detection thresholds and thermal sensitivity remained largely stable, vibration detection thresholds and pressure pain thresholds demonstrated longitudinal changes. VDT alterations were site-specific and occurred on both the affected and non-affected sides, with more pronounced changes at distal anatomical locations. These findings are consistent with broader alterations in sensory processing that may reflect the combined influence of surgery, adjuvant treatments, recovery processes and individual adaptation mechanisms rather than isolated local effects [30,75,76,77]. However, given the observational design and heterogeneous treatment pathways, causal attribution to specific oncological therapies cannot be established.

Reduced pressure pain thresholds observed during follow-up are consistent with previous studies reporting lower thresholds after breast cancer surgery [35,77] and associations with pain presence and intensity among breast cancer survivors [33]. These findings support the potential value of pressure pain thresholds as indicators of altered pain sensitivity. Nevertheless, the variability observed across muscles and assessment points suggests that sensory recovery may follow heterogeneous and non-linear trajectories rather than a single directional pattern [30]. Accordingly, these findings should be interpreted as evidence of dynamic sensory adaptation rather than a uniform mechanistic pathway.

Previous studies have reported increased thermal perception thresholds and MDT alterations following surgery [29,31,71,78], with more pronounced impairments after mastectomy and axillary lymph node dissection compared with breast-conserving surgery or sentinel node biopsy [79]. Similar findings have been reported following chemotherapy and in patients with treatment-related neuropathy. In contrast, the relative stability of these measures in the present cohort may reflect differences in surgical techniques, adjuvant-treatment exposure or assessment timing.

Temporal summation declined over time whereas CPM responses deteriorated, characterized by increased conditioning pain intensity and reduced post-conditioning pressure pain thresholds. This apparent dissociation underscores the complexity of pain modulation during survivorship recovery and suggests that facilitatory and inhibitory mechanisms may evolve differently over time rather than reflecting a single underlying process. Similar dissociations have been reported in other chronic pain and cancer-related pain populations [22,63,80]. Rather than indicating recovery from central sensitization, reduced temporal summation may reflect adaptive or compensatory changes in spinal nociceptive processing [35,63,75], particularly in a cohort characterized by relatively low pain intensity and CSI scores consistently below the clinical threshold. In contrast, CPM may be more sensitive to contextual and psychological influences [81,82], underscoring the multifactorial nature of pain modulation following cancer treatment.

Self-reported symptoms of central sensitization remained below the clinical threshold throughout follow-up despite objective sensory alterations detected by QST. This dissociation between objective sensory alterations and subjective symptom perception suggests that sensory changes do not necessarily translate into clinically perceived symptom worsening [83]. Consistently low levels of pain catastrophizing may partly explain the relative stability of symptom perception despite objective sensory changes, highlighting the value of combining objective and self-reported measures when evaluating recovery after breast cancer treatment.

From a clinical perspective, these findings suggest that survivorship assessment based solely on pain intensity may underestimate ongoing recovery-related difficulties. Incorporating psychosocial outcomes, HRQoL measures and selected sensory assessments may help identify women at risk of persistent dysfunction despite relatively low symptom severity. Such multidimensional approaches may facilitate more personalized rehabilitation and supportive care strategies throughout breast cancer survivorship.

### Strengths and Limitations

To our knowledge, this is the first study to longitudinally evaluate sensory trajectories across multiple stages of breast cancer treatment up to 12 months after surgery, integrating QST with psychosocial, functional and HRQoL outcomes. The study additionally provides a multidimensional perspective on survivorship recovery by capturing sensory, psychosocial and quality-of-life changes throughout the treatment continuum. The longitudinal design enabled the identification of dynamic recovery patterns and temporal divergences between objective and subjective outcomes, enhancing the ecological validity and clinical relevance of the findings.

Several limitations should nevertheless be acknowledged. First, the single-centre design may limit generalizability.

Second, participant attrition was substantial, with 43 of 72 participants completing the 12-month assessment. Baseline comparisons suggested potential selective attrition, as non-completers reported greater pain intensity during the conditioning stimulus and poorer HRQoL before starting the oncological treatment pathway. However, these differences cannot establish whether these baseline characteristics influenced subsequent follow-up completion, and reasons for dropout were not systematically retrieved. Although linear mixed-effects models incorporated all available data under a missing-at-random assumption, this assumption cannot be empirically verified. Therefore, attrition may have influenced some estimated longitudinal changes.

Third, while QST and the Central Sensitization Inventory provide valuable insights into pain-related sensory processing and sensitization, both measures have inherent limitations and should not be considered direct biomarkers of underlying neurophysiological mechanisms. Furthermore, although analyses were adjusted for relevant confounders, unmeasured factors such as fatigue, physical activity, sleep quality or cognitive function may have influenced recovery trajectories. Pain-related medication use was not systematically recorded, which represents an important limitation when interpreting pain-related outcomes and CPM. Analgesic use may reduce perceived pain intensity, potentially masking changes in the underlying pain experience, while pharmacological modulation of descending pain pathways may also influence CPM responses. Consequently, the relative stability of pain intensity observed throughout follow-up may partly reflect effective but unmeasured analgesic use, potentially leading to an underestimation of changes in pain severity. Similarly, unmeasured medication use may have contributed to the observed variability in CPM, limiting the extent to which longitudinal changes can be attributed to changes in endogenous pain modulation alone.

In addition, the broad multidimensional assessment resulted in multiple longitudinal comparisons. Family-wise error was controlled for the principal outcome family underpinning the sample-size calculation; however, the remaining outcomes were exploratory and were not individually powered or adjusted for multiplicity. Furthermore, the a priori sample-size calculation was based specifically on CPM/PPT-related measures and therefore did not provide outcome-specific power for these exploratory outcomes. Estimates for these outcomes should consequently be interpreted cautiously, considering the magnitude and precision of the observed changes, and as hypothesis-generating rather than definitive evidence of longitudinal change, particularly where isolated changes were observed without a consistent longitudinal pattern.

Finally, participants followed heterogeneous treatment pathways, and the nominal assessment phases did not necessarily represent the same clinical event or exact calendar time for all participants, particularly during intermediate follow-up. Although chemotherapy and radiotherapy exposure were included as covariates in the longitudinal analyses, this adjustment cannot fully account for differences in the timing or clinical meaning of individual assessment points. The longitudinal effects should therefore be interpreted as average changes across predefined follow-up phases rather than treatment-specific trajectories. A sensitivity analysis restricted to participants undergoing surgery alone was considered; however, this subgroup comprised only 25 participants at baseline and was further reduced across follow-up, with only seven contributing data at the final assessment. Such an analysis would therefore have produced highly imprecise and potentially unstable longitudinal estimates. Future adequately powered, treatment-stratified studies are needed to characterize treatment-specific trajectories.

## 5. Conclusions

Recovery after breast cancer treatment was characterized by heterogeneous sensory, psychosocial and health-related quality-of-life trajectories that were not consistently reflected by pain severity alone. Although pain intensity remained relatively stable, kinesiophobia increased and HRQoL deteriorated over time, suggesting that important survivorship challenges may persist despite partial physical recovery.

Objective sensory alterations additionally suggest evolving changes in somatosensory processing throughout survivorship. Together, these findings support the view that recovery after breast cancer treatment is multidimensional and extends beyond pain reduction or isolated functional outcomes.

Integrating sensory assessment, psychosocial evaluation and patient-reported outcomes may help identify women at risk of persistent recovery-related difficulties and facilitate more personalized rehabilitation and supportive care strategies. Future research should investigate the prognostic value of sensory alterations, the contribution of specific oncological therapies and the effectiveness of targeted interventions, including exercise, pain education, behavioural approaches and neuromodulation, to optimize survivorship care.

## Figures and Tables

**Table 1:** Baseline characteristics of women with breast cancer undergoing oncological treatment.

	All
Subject, n	72
Age, years mean ± SD	57 ± 11
Body mass index, kg·m^−2^ mean ± SD	27.5 ± 5.6
Oncological treatment	
Surgery	
Side	
Right n (%)	40 (56)
Left n (%)	32 (44)
Breast surgery	
Lumpectomy n (%)	29 (40)
Unilateral mastectomy n (%)	14 (19)
Quadrantectomy n (%)	3 (4)
Data unavailable * n (%)	18 (25)
Axillary surgery	
Selective sentinel lymph node biopsy n (%)	40 (56)
Lymphadenectomy n (%)	5 (7)
Data unavailable * n (%)	16 (22)
Lymph nodes removed	
Less than 10 nodes n (%)	33 (46)
10–20 nodes n (%)	2 (3)
Adjuvant treatments	
Chemotherapy n (%)	15 (21)
Chemotherapy sessions, n median (Q1;Q3)	8 (6;14)
Radiotherapy n (%)	34 (47)
Radiotherapy sessions, n median (Q1;Q3)	15 (15;15)
Hormone therapy n (%)	44 (61)
Biological therapy n (%)	7 (10)

Continuous variables are presented as mean ± SD or median (Q1;Q3), as appropriate, and categorical variables as n (%). * Data unavailable: the specific surgical procedure was not documented in the available clinical records and could not be reliably ascertained from participant-reported information.

**Table 2:** (**A**) Longitudinal changes in primary/confirmatory quantitative sensory testing outcomes in women undergoing breast cancer treatment. (**B**) Longitudinal changes in exploratory quantitative sensory testing outcomes in women undergoing breast cancer treatment.

**(A)**								
	**A0**	**A1**	**A2**	**A3**	**A4**	**A5**	**Overall Time Effect, *p***	**Holm-Adjusted *p***
Participants, n	72	64	58	51	45	43		
Non-affected scalene PPT post, kilogrammes	1.78 ± 0.33	2.06 ± 0.28	1.42 ± 0.18	1.36 ± 0.17	1.95 ± 0.14	1.61 ± 0.59	*p* < 0.001	*p* < 0.001
CPM, NRS 0–10	3.00 ± 0.81	5.00 ± 0.83	3.14 ± 0.77	5.80 ± 0.62	7.12 ± 0.78	7.19 ± 1.12	*p* = 0.004	*p* = 0.031
Non-affected side rectus femoris CPM ΔPPT, kilogrammes	4.75 ± 0.56	3.90 ± 0.66	3.45 ± 0.53	2.84 ± 0.44	3.28 ± 0.30	3.92 ± 0.53	*p* = 0.005	*p* = 0.031
Non-affected serratus PPT post, kilogrammes	2.36 ± 0.32	2.57 ± 0.21	2.29 ± 0.19	2.50 ± 0.26	1.64 ± 0.22	2.18 ± 0.38	*p* = 0.010	*p* = 0.048
Affected side infraspinatus CPM ΔPPT, kilogrammes	3.98 ± 0.77	1.97 ± 0.42	2.43 ± 0.40	3.08 ± 0.56	2.80 ± 0.28	2.53 ± 0.31	*p* = 0.202	*p* = 0.808
Non-affected epicondyles PPT post, kilogrammes	2.99 ± 0.59	2.76 ± 0.29	2.39 ± 1.51	2.53 ± 0.16	2.31 ± 0.12	3.06 ± 1.08	*p* = 0.231	*p* = 0.808
Non-affected side infraspinatus CPM ΔPPT, kilogrammes	3.65 ± 0.84	2.84 ± 0.41	2.58 ± 0.31	3.36 ± 0.70	3.01 ± 0.30	2.31 ± 0.47	*p* = 0.471	*p* = 0.942
Non-affected vastus medialis PPT post, kilogrammes	3.74 ± 0.48	3.67 ± 0.41	3.72 ± 0.21	3.56 ± 0.48	3.58 ± 0.36	3.29 ± 0.76	*p* = 0.781	*p* = 0.942
(**B**)								
	**A0**	**A1**	**A2**	**A3**	**A4**	**A5**	**Overall Time Effect, *p***	
Participants, n	72	64	58	51	45	43		
Affected side, increasing MDT diameter, grammes	0.346 ± 0.080	0.384 ± 0.146	0.405 ± 0.115	0.225 ± 0.100	0.247 ± 0.039	0.369 ± 0.086	*p* = 1.000	
Affected side, decreasing MDT diameter, grammes	0.355 ± 0.168	2.115 ± 1.549	0.330 ± 0.181	0.186 ± 0.088	0.135 ± 0.042	1.732 ± 1.219	*p* = 1.000	
Non-affected side, increasing MDT diameter, grammes	0.322 ± 0.114	0.389 ± 0.118	0.281 ± 0.125	0.311 ± 0.088	0.311 ± 0.046	0.427 ± 0.075	*p* = 0.075	
Non-affected side, decreasing MDT diameter, grammes	0.349 ± 0.176	0.363 ± 0.175	0.317 ± 0.177	0.285 ± 0.088	0.224 ± 0.062	0.145 ± 0.044	*p* = 1.000	
Affected side acromion VDT, seconds	12.76 ± 1.09	9.41 ± 0.73	10.68 ± 1.11	12.17 ± 1.31	12.94 ± 2.18	10.77 ± 1.49	*p* < 0.001	
Affected side epicondyles VDT, seconds	13.48 ± 2.48	9.31 ± 1.07	10.27 ± 1.56	12.59 ± 1.76	16.85 ± 1.32	3.80 ± 5.54	*p* < 0.001	
Affected side styloid VDT, seconds	13.91 ± 1.35	15.23 ± 1.32	15.05 ± 1.91	15.20 ± 1.99	14.56 ± 3.31	19.99 ± 1.38	*p* < 0.001	
Non-affected side acromion VDT, seconds	10.84 ± 1.45	11.12 ± 1.54	10.18 ± 1.33	10.39 ± 1.51	12.81 ± 1.66	10.17 ± 1.74	*p* = 0.035	
Non-affected side epicondyles VDT, seconds	11.98 ± 1.69	10.00 ± 0.65	10.17 ± 0.53	13.04 ± 1.21	18.34 ± 1.27	13.22 ± 2.32	*p* < 0.001	
Non-affected side styloid VDT, seconds	13.29 ± 1.05	15.73 ± 1.44	15.83 ± 1.63	18.39 ± 1.77	16.09 ± 3.08	17.95 ± 2.67	*p* < 0.001	
Affected side TS, wind-up ratio	1.44 ± 0.21	1.56 ± 0.32	1.52 ± 0.20	1.37 ± 0.11	1.26 ± 0.09	1.31 ± 0.19	*p* < 0.001	
Non-affected side TS, wind-up ratio	1.80 ± 0.31	2.08 ± 0.91	1.63 ± 0.28	1.34 ± 0.13	1.33 ± 0.11	1.31 ± 0.13	*p* = 0.509	
STPS location (local/referred) n (%)	58 (81)/8 (11)	56 (88)/5 (8)	47 (81)/4 (7)	35 (69)/5 (10)	31 (69)/6 (9)	30 (70)/2 (5)	-	
STPS, NRS 0–10	7.24 ± 0.84	2.59 ± 1.32	4.43 ± 0.62	6.03 ± 0.92	4.92 ± 0.89	5.71 ± 0.94	*p* = 0.419	

Values are presented as estimated marginal mean ± SE, from linear mixed-effects models. Overall time effects were assessed using linear mixed-effects models, with time as a fixed effect and participant as a random effect, adjusted for age, baseline body mass index, number of chemotherapy cycles, and number of radiotherapy sessions. Variation in sample size across time points reflects loss to follow-up and was accounted for by the mixed-model approach. Unadjusted omnibus *p*-values and Holm-adjusted *p*-values are reported. Holm adjustment was applied across the principal outcome family to control the family-wise Type I error rate at α = 0.05; Holm-adjusted *p* < 0.05 was considered statistically significant. Pairwise comparisons, where performed, were Bonferroni-adjusted, with adjusted *p* < 0.05 considered statistically significant. Abbreviations: A0: baseline/before surgery; A1: early post-surgery (1 week after surgery); A2: pre-adjuvant (1 month after surgery/~before radiotherapy/~before chemotherapy); A3: mid-adjuvant (3 months after surgery/~after radiotherapy/~6 months after surgery ~after chemotherapy ~before radiotherapy); A4: late-adjuvant (6 months after surgery/~9 months after surgery ~after radiotherapy); A5: 12 months after surgery; CPM: conditioned pain modulation; NRS: numerical rating scale; PPT: pressure pain threshold. Values are presented as estimated marginal mean ± SE, from linear mixed-effects models, unless otherwise reported. Overall time effects were assessed using linear mixed-effects models, with time as a fixed effect and participant as a random effect, adjusted for age, baseline body mass index, number of chemotherapy cycles, and number of radiotherapy sessions. Variation in sample size across time points reflects loss to follow-up and was accounted for by the mixed-model approach. Overall time-effect *p*-values are reported unadjusted for multiplicity across outcomes and should be interpreted as hypothesis-generating rather than according to a formal significance threshold. Abbreviations: A0: baseline/before surgery; A1: early post-surgery (1 week after surgery); A2: pre-adjuvant (1 month after surgery/~before radiotherapy/~before chemotherapy); A3: mid-adjuvant (3 months after surgery/~after radiotherapy/~6 months after surgery ~after chemotherapy ~before radiotherapy); A4: late-adjuvant (6 months after surgery/~9 months after surgery ~after radiotherapy); A5: 12 months after surgery; MDT: mechanical detection threshold; NRS: numerical rating scale; STPS: supra-threshold pressure stimulation; TS: temporal summation; VDT: vibration detection threshold.

**Table 3:** Longitudinal changes in exploratory patient-reported outcomes of women undergoing breast cancer treatment: pain, sensitization indicators, psychological factors, and health-related quality of life.

	A0	A1	A2	A3	A4	A5	Overall Time Effect (*p*-Value)
Participants, n	72	64	58	51	45	43	
Symptoms and health-related quality of life							
CSI, total score ^b^	27 (20;36)	24 (13;36)	22 (13;32)	27 (14;37)	25 (13;43)	23 (14;37)	*p* = 0.862
PCS, total score ^b^	15 (7;23)	11 (4;20)	13 (2;18)	8 (2;19)	16 (7;25)	12 (3;23)	*p* = 0.108
TSK, total score ^a^	15 ± 3	16 ± 2	16 ± 2	19 ± 3	16 ± 2	24 ± 4	*p* = 0.002
FACT-B, total score ^a^	114 ± 6	108 ± 6	114 ± 6	109 ± 8	113 ± 7	100 ± 10	*p* < 0.001
FACT-B, TOI score ^a^	71 ± 4	70 ± 3	71 ± 3	68 ± 5	70 ± 5	60 ± 7	*p* = 0.959
FACT-G, total score ^b^	85 (71;92)	85 (70;94)	88 (76;94)	87 (78;93)	86 (71;96)	87 (76;95)	*p* = 0.844
Participants reporting pain, n	16	26	18	17	14	12	
BPI, pain severity score 0–10 ^b^	4 (2;5)	3 (1;5)	3 (0;4)	4 (2;5)	4 (3;5)	5 (1;7)	*p* = 0.255
BPI, interpretation ^c^ (mild/moderate/severe) n (%)	12 (17)/2 (3)/2 (3)	19 (30)/2 (3)/5 (8)	16 (28)/2 (3)/0 (0)	10 (20)/6 (12)/1 (2)	10 (22)/3 (7)/1 (2)	6 (14)/4 (9)/2 (5)	-
SPADI, pain score ^b^	22 (6;34)	16 (5;28)	23 (11;28)	18 (7;34)	21 (4;29)	15 (3;18)	*p* = 0.416
SPADI, disability score ^b^	19 (7;38)	29 (7;52)	22 (3;45)	14 (5;28)	27 (13;42)	11 (7;26)	*p* = 0.416
SPADI, total score ^b^	26 (8;75)	33 (11;73)	49 (17;69)	43 (14;64)	44 (18;74)	25 (14;56)	*p* = 0.255
S-LANSS, total score ^b^	9 (6;13)	8 (5;15)	8 (3;11)	12 (7;16)	14 (3;16)	6 (3;9)	-
CPAQ, total score ^b^	63 (29;74)	69 (58;78)	59 (37;78)	59 (46;73)	62 (44;82)	64 (19;77)	*p* = 0.335

Values are expressed as mean ± SE or median (Q1;Q3), as appropriate, unless otherwise reported. Overall time effects were assessed using linear mixed-effects models and Friedman tests for variables with normally and non-normally distributed residuals, respectively. Data from linear mixed-effects models were reported as estimated marginal mean ± SE. ^a^ Linear mixed-effects model; ^b^ Friedman test; ^c^ Cochran’s Q. Overall time-effect *p*-values are reported unadjusted for multiplicity across outcomes and should be interpreted as hypothesis-generating rather than according to a formal significance threshold. Abbreviations: A0: baseline/before surgery; A1: early post-surgery (1 week after surgery); A2: pre-adjuvant (1 month after surgery/~before radiotherapy/~before chemotherapy); A3: mid-adjuvant (3 months after surgery/~after radiotherapy/~6 months after surgery ~after chemotherapy ~before radiotherapy); A4: late-adjuvant (6 months after surgery/~9 months after surgery ~after radiotherapy); A5: 12 months after surgery; CPAQ: Chronic Pain Acceptance Questionnaire; CSI: Central Sensitization Inventory; FACT-B: Functional Assessment of Cancer Therapy-Breast; FACT-G: Functional Assessment of Cancer Therapy-General; S-LANSS: Leeds Assessment of Neuropathic Symptoms and Signs; PCS: Pain Catastrophizing Scale; SPADI: Shoulder Pain and Disability Index; TSK: Tampa Scale of Kinesiophobia.

## Data Availability

The data presented in this study are available on request from the corresponding author due to privacy and ethical restrictions related to the protection of participants’ confidential data.

## Supplementary Materials

The following supporting information can be downloaded at https://www.mdpi.com/article/10.3390/curroncol33090567/s1, Figure S1: Quantitative sensory testing—Mechanical detection threshold in women undergoing breast cancer treatment over 12 months of follow-up; Figure S2: Quantitative sensory testing—Vibration detection threshold in women undergoing breast cancer treatment over 12 months of follow-up; Figure S3: Quantitative sensory testing—Temporal summation in women undergoing breast cancer treatment over 12 months of follow-up; Figure S4: Quantitative sensory testing—Pressure pain threshold in women undergoing breast cancer treatment over 12 months of follow-up; Figure S5: Quantitative sensory testing—Conditioned pain modulation in women undergoing breast cancer treatment over 12 months of follow-up; Figure S6: Quantitative sensory testing—Supra-threshold pressure stimulation in women undergoing breast cancer treatment over 12 months of follow-up; Table S1: Baseline characteristics and outcome measures of completers vs. non-completers among women treated for breast cancer undergoing longitudinal follow-up; Table S2: Actual calendar timing of assessments relative to breast cancer surgery.

## Abbreviations

BMI	Body Mass Index
BPI	Brief Pain Inventory
CPM	Conditioned Pain Modulation
CPAQ	Chronic Pain Acceptance Questionnaire
CSI	Central Sensitization Inventory
FACT-B	Functional Assessment of Cancer Therapy-Breast
HRQoL	Health-Related Quality of Life
MDT	Mechanical Detection Threshold
PCS	Pain Catastrophizing Scale
PPT	Pressure Pain Threshold
QST	Quantitative Sensory Testing
SPADI	Shoulder Pain and Disability Index
S-LANSS	Self-report Leeds Assessment of Neuropathic Symptoms and Signs
TSK-11	Tampa Scale of Kinesiophobia (11-Item Version)
TS	Temporal Summation
VDT	Vibration Detection Threshold
WUR	Wind-Up Ratio
