# Supplementary material for: Multidimensional Recovery Trajectories Throughout Breast Cancer Treatment and Survivorship: A Prospective Longitudinal Study
Source: Curr Oncol. 2026 Sep 19;33(9):567. doi: 10.3390/curroncol33090567 (PMC13605617; doi:10.3390/curroncol33090567)
Supplement: Supplementary file 1 [file curroncol-33-00567-s001.zip › curroncol-4518091-supplementary.pdf]

Title: Multidimensional recovery trajectories throughout breast cancer treatment and survivorship: a prospective longitudinal study

Authors: Laura Lorenzo-Gallego, Nicola S. Diciolla, Fernando Ramos-Gómez, Virginia Prieto-Gómez, Aldina Couso González, María Torres-Lacomba.

Corresponding author details:
Name and surname: Nicola S. Diciolla
Email: nicola.diciolla@uah.es
Contact address: Physiotherapy in Women's Health Research Group—FPSM, Department of Nursing and Physiotherapy, University of Alcalá, Alcalá de Henares, 28805 Madrid, Spain. Tel: +34 918 854 828

Supplementary materials

Contents

Section 1) Results 2

Tables 2

Table S1. Baseline characteristics and outcome measures of completers vs. non-completers among women treated for breast cancer undergoing longitudinal follow-up 2

Table S2. Actual calendar timing of assessments relative to breast cancer surgery 3

Figures 4

Figure S1. Quantitative sensory testing – mechanical detection threshold in women treated for breast cancer over twelve-months follow-up 4

Figure S2. Quantitative sensory testing – vibration detection threshold in women treated for breast cancer over twelve-months follow-up 5

Figure S3. Quantitative sensory testing – temporal summation in women treated for breast cancer over twelve-months follow-up 7

Figure S4. Quantitative sensory testing – pressure pain threshold in women treated for breast cancer over twelve-months follow-up 8

Figure S5. Quantitative sensory testing – conditioned pain modulation in women treated for breast cancer over twelve-months follow-up 9

Figure S6. Quantitative sensory testing – supra-threshold pressure stimulation in women treated for breast cancer over twelve-months follow-up 10

Section 1) Results
Tables
Table S1. Baseline characteristics and outcome measures of completers vs. non-completers among women treated for breast cancer undergoing longitudinal follow-up

	All	Non-completers	Completers	Between-group difference[95%CI]
**Subject, n**	**72**	**29**	**43**	
Age, years mean±SD	57±11	59±11	55±10	3.69[-1.58;8.98]
Body mass index, kg·m^{-2} mean±SD	27.5±5.6	28.5±5.7	26.3±5.1	2.20[-0.50;5.06]
**Quantitative sensory testing outcomes**				
Not affected scalene PPT post, kg	1.49±0.42	1.48±0.46	1.49±0.85	-0.01[-0.39;0.32]
Not affected serratus PPT post, kg	2.46±0.90	2.48±0.84	2.40±0.92	0.08[-0.36;0.52]
Not affected epicondyles PPT post, kg	2.43±0.59	2.48±0.68	2.56±0.50	-0.08[-0.60;0.37]
Not affected vastus medialis PPT post, kg	3.76±1.43	3.64±1.53	3.85±1.39	-0.21[-0.95;0.53]
**20 repetitions CPM, NRS 0-10**	**5.13±2.89**	**5.72±2.88**	**4.05±2.83**	**1.67[0.26;3.06]**
Affected side infraspinatus CPM PPT, kg	3.29±1.36	2.98±1.22	3.43±2.02	-0.45[-1.28;0.35]
Not affected side infraspinatus CPM PPT, kg	3.23±1.39	3.00±1.37	3.53±1.89	-0.52[-1.36;0.28]
Not affected side rectus femoris CPM PPT, kg	4.06±1.59	3.97±1.75	4.39±1.70	-0.43[-1.31;0.41]
**Symptoms and health-related quality of life**				
CSI, total score	28(22;37)	34(26;51)	27(22;35)	4 [-2;13]
PCS, total score	15(7;24)	15(5;25)	16(8;23)	-2 [-9;5]
TSK, total score	22(16;26)	22(18;26)	22(16;26)	3 [-1;6]
**FACT-B, total score**	**109(95;121)**	**95(78;113)**	**113(103;123)**	**-13 [-22;-4]**
**FACT-B, TOI score**	**70(59;77)**	**65(53;74)**	**72(63;79)**	**-7[-14;-2]**
**FACT-G, total score**	**85(71;92)**	**69(51;86)**	**88(80;93)**	**-13[-21;-4]**
**Pain**	**16**			
BPI, pain severity score 0-10	5(3;6)	4(1;5)	5(4;7)	1 [-2;4]
SPADI, pain score	34(8;40)	35(9;45)	27(8;40)	-3[-20;13]
SPADI, disability score	23(16;47)	35(9;69)	23(16;39)	0[-18;33]
SPADI, total score	74(24;91)	91(43;115)	26(13;79)	5[-19;63]
S-LANSS, total score	6(2;14)	4(1;8)	11(4;16)	-5[-10;2]
CPAQ, total score	71(45;77)	67(45;75)	71(29;84)	5[-17;40]

Data are presented as mean±SD or median(Q1;Q3), as appropriate.

Between-group differences were calculated as non-completers minus completers. For approximately normally distributed continuous variables, unadjusted mean differences with 95% bias-corrected and accelerated bootstrap confidence intervals based on 5000 resamples are reported. For non-normally distributed continuous variables, Hodges–Lehmann estimates of the between-group location shift with 95% confidence intervals are reported.

Positive estimates indicate higher values among non-completers and negative estimates indicate lower values among non-completers.

Abbreviations: BPI: Brief Pain Inventory; CPAQ: Chronic Pain Acceptance Questionnaire; CPM: conditioned pain modulation; CSI: Central Sensitisation Inventory; FACT-B: Functional Assessment of Cancer Therapy–Breast; FACT-G: Functional Assessment of Cancer Therapy–General; NRS: numerical rating scale; PCS: Pain Catastrophizing Scale; PPT: pressure pain threshold; S-LANSS: Self-report Leeds Assessment of Neuropathic Symptoms and Signs; SPADI: Shoulder Pain and Disability Index; TOI: Trial Outcome Index; TSK: Tampa Scale of Kinesiophobia.

Table S2. Actual calendar timing of assessments relative to breast cancer surgery

Assessment	n	Nominal timing	Day relative to surgery median(Q1;Q3)	Range, min-max
**A0**	72	Before surgery	-7(-7;-7)	-7 to -7
**A1**	64	~1 week	7(7;7)	7-7
**A2**	58	~1 month	30(30;30)	30-30
**A3**	51	~3 months	90(90;90)	90-240
**A4**	45	~6 months	201(187;208)	180-330
**A5**	43	~12 months	381(367;395)	360-510

Negative values indicate assessments performed before surgery; positive values indicate assessments performed after surgery.

Abbreviations: A0: Baseline/before surgery; A1: Early post-surgery (1 week after surgery); A2: Pre-adjuvant (1 month after surgery/ ~before radiotherapy/ ~before chemotherapy); A3: Mid-adjuvant (3 months after surgery/ ~after radiotherapy/ ~6 months after surgery ~after chemotherapy ~before radiotherapy); A4: Late-adjuvant (6 months after surgery/ ~9 months after surgery ~after radiotherapy); A5: 12 months after surgery.

Figures

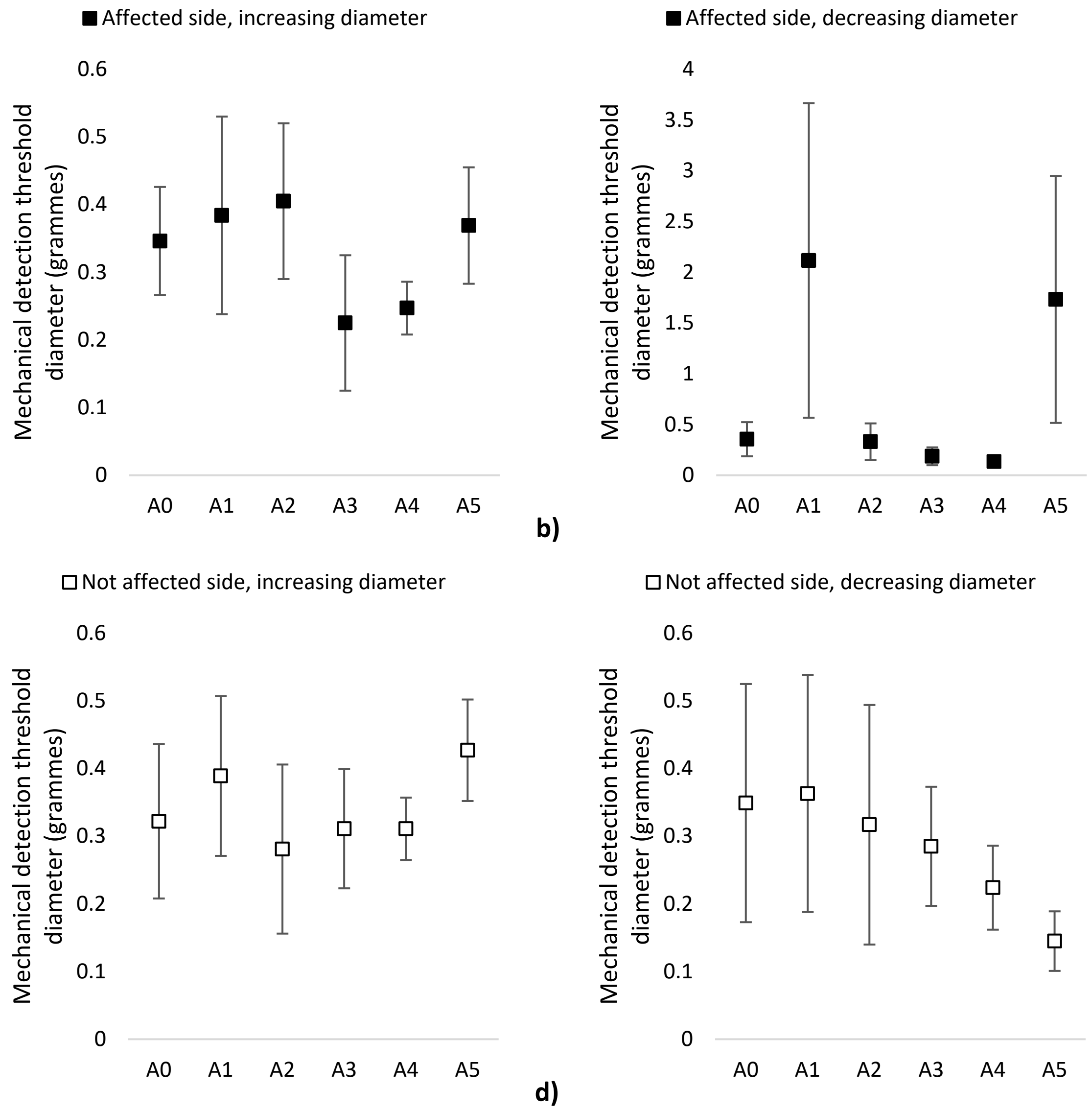

Figure S1. Quantitative sensory testing – mechanical detection threshold in women treated for breast cancer over twelve-months follow-up

Abbreviations: A0: Baseline/before surgery; A1: Early post-surgery (1 week after surgery); A2: Pre-adjuvant (1 month after surgery/ ~before radiotherapy/ ~before chemotherapy); A3: Mid-adjuvant (3 months after surgery/ ~after radiotherapy/ ~6 months after surgery ~after chemotherapy ~before radiotherapy); A4: Late-adjuvant (6 months after surgery/ ~9 months after surgery ~after radiotherapy); A5: 12 months after surgery.

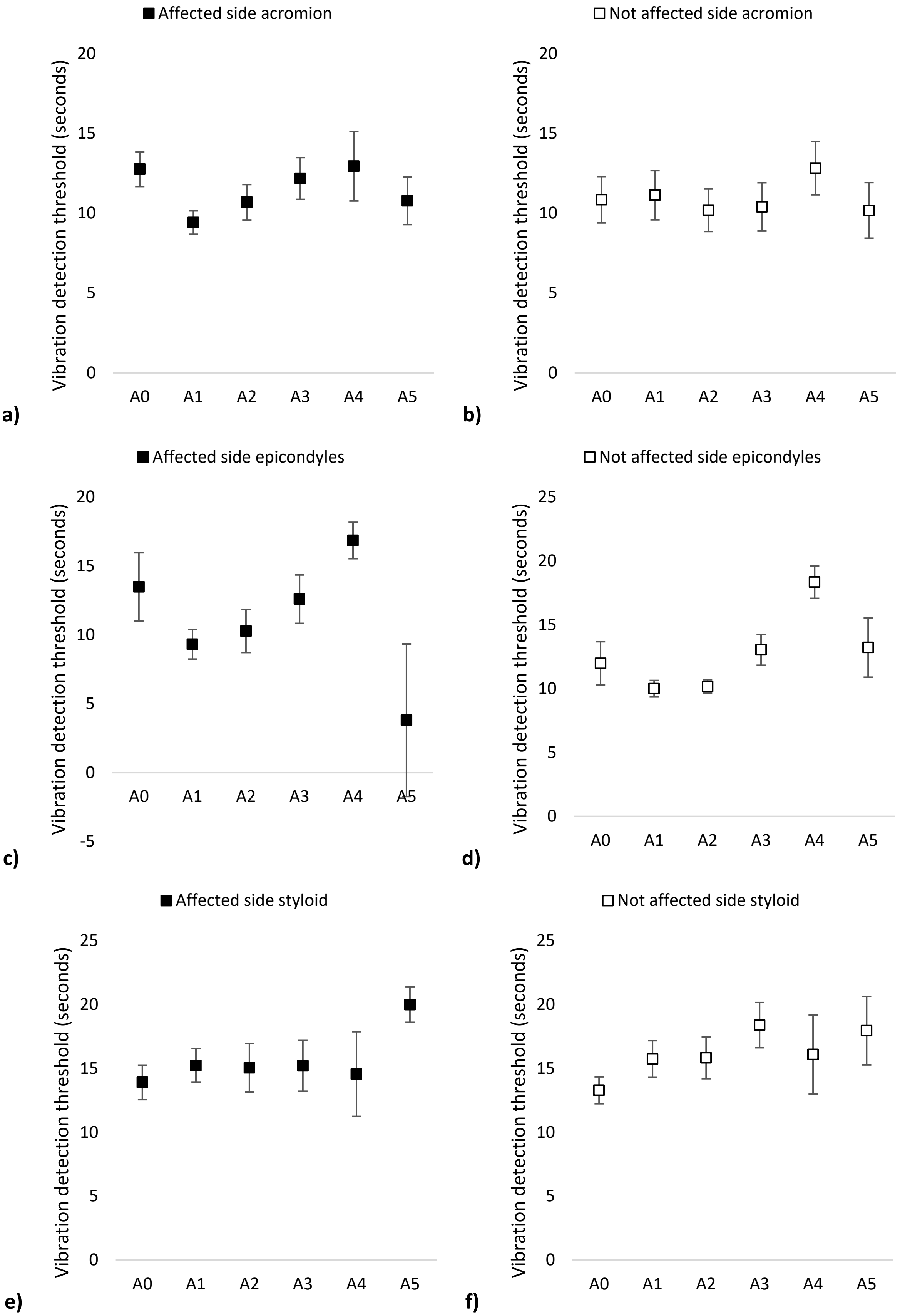

Figure S2. Quantitative sensory testing – vibration detection threshold in women treated for breast cancer over twelve-months follow-up

Abbreviations: A0: Baseline/before surgery; A1: Early post-surgery (1 week after surgery); A2: Pre-adjuvant (1 month after surgery/ ~before radiotherapy/ ~before chemotherapy); A3: Mid-adjuvant (3 months after

surgery/ ~after radiotherapy/ ~6 months after surgery ~after chemotherapy ~before radiotherapy); A4: Late-adjuvant (6 months after surgery/ ~9 months after surgery ~after radiotherapy); A5: 12 months after surgery.

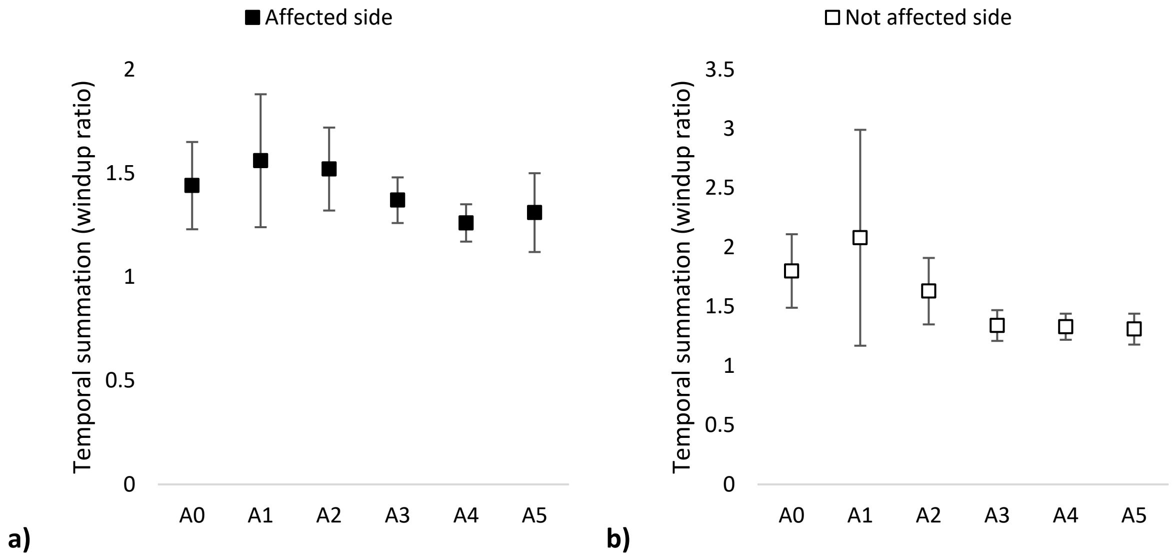

Figure S3. Quantitative sensory testing – temporal summation in women treated for breast cancer over twelve-months follow-up

Abbreviations: A0: Baseline/before surgery; A1: Early post-surgery (1 week after surgery); A2: Pre-adjuvant (1 month after surgery/ ~before radiotherapy/ ~before chemotherapy); A3: Mid-adjuvant (3 months after surgery/ ~after radiotherapy/ ~6 months after surgery ~after chemotherapy ~before radiotherapy); A4: Late-adjuvant (6 months after surgery/ ~9 months after surgery ~after radiotherapy); A5: 12 months after surgery.

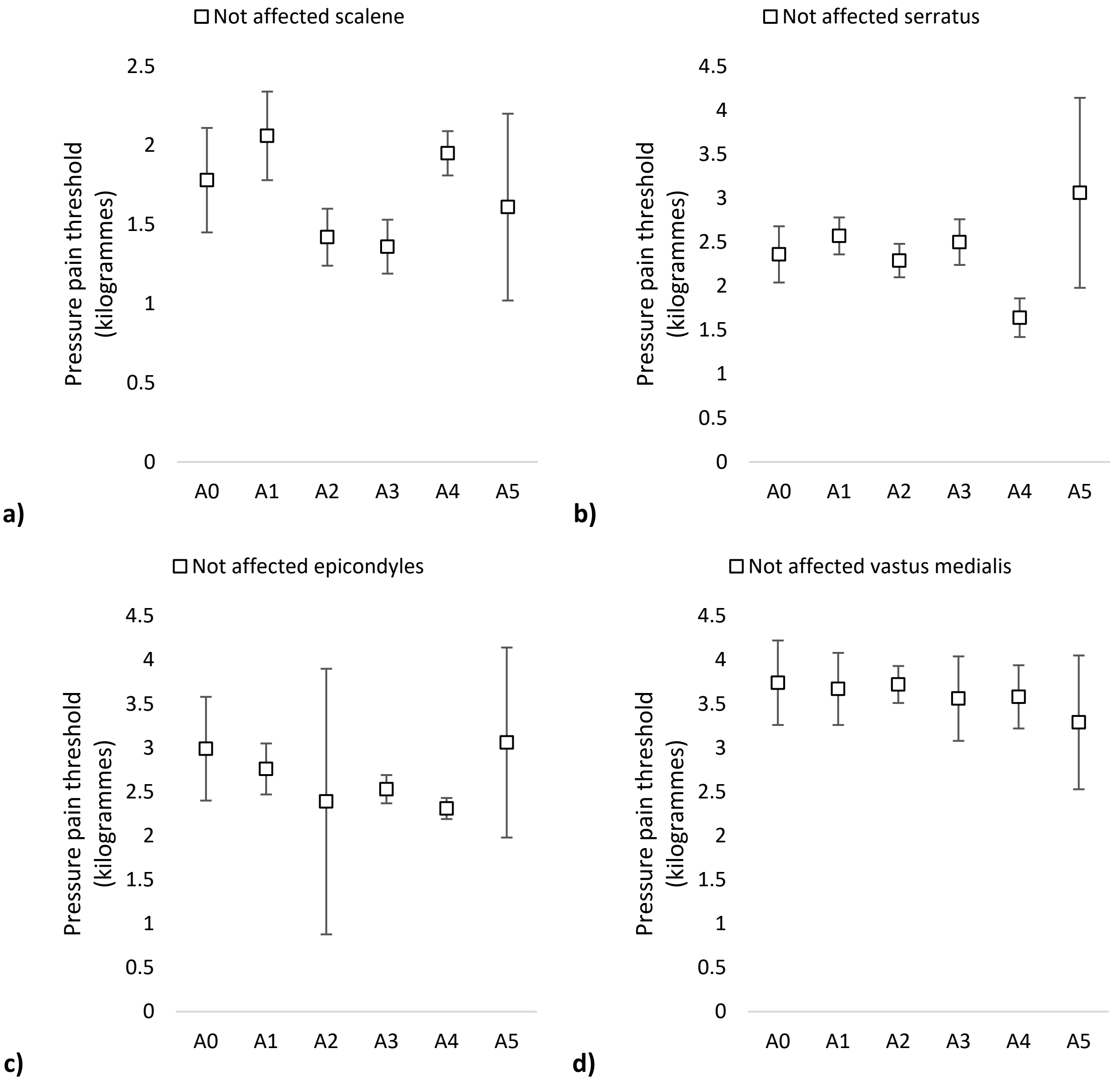

Figure S4. Quantitative sensory testing – pressure pain threshold in women treated for breast cancer over twelve-months follow-up

Abbreviations: A0: Baseline/before surgery; A1: Early post-surgery (1 week after surgery); A2: Pre-adjuvant (1 month after surgery/ ~before radiotherapy/ ~before chemotherapy); A3: Mid-adjuvant (3 months after surgery/ ~after radiotherapy/ ~6 months after surgery ~after chemotherapy ~before radiotherapy); A4: Late-adjuvant (6 months after surgery/ ~9 months after surgery ~after radiotherapy); A5: 12 months after surgery.

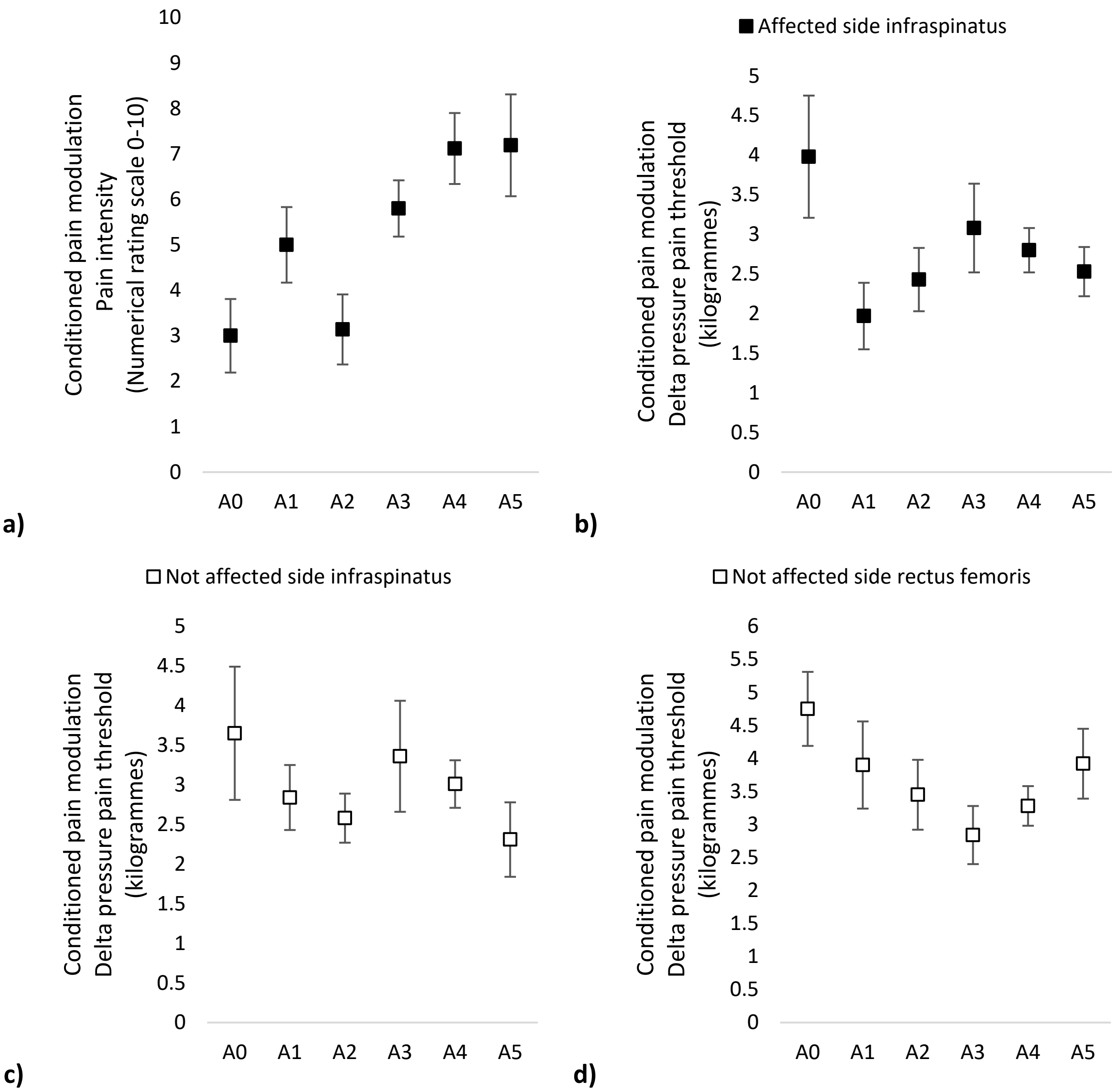

Figure S5. Quantitative sensory testing – conditioned pain modulation in women treated for breast cancer over twelve-months follow-up

Abbreviations: A0: Baseline/before surgery; A1: Early post-surgery (1 week after surgery); A2: Pre-adjuvant (1 month after surgery/ ~before radiotherapy/ ~before chemotherapy); A3: Mid-adjuvant (3 months after surgery/ ~after radiotherapy/ ~6 months after surgery ~after chemotherapy ~before radiotherapy); A4: Late-adjuvant (6 months after surgery/ ~9 months after surgery ~after radiotherapy); A5: 12 months after surgery.

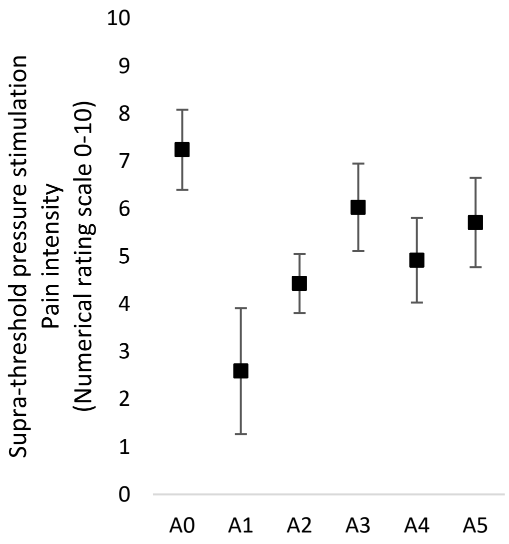

Figure S6. Quantitative sensory testing – supra-threshold pressure stimulation in women treated for breast cancer over twelve-months follow-up

Abbreviations: A0: Baseline/before surgery; A1: Early post-surgery (1 week after surgery); A2: Pre-adjuvant (1 month after surgery/ ~before radiotherapy/ ~before chemotherapy); A3: Mid-adjuvant (3 months after surgery/ ~after radiotherapy/ ~6 months after surgery ~after chemotherapy ~before radiotherapy); A4: Late-adjuvant (6 months after surgery/ ~9 months after surgery ~after radiotherapy); A5: 12 months after surgery.
